# Supplementary material for: Optimal free descriptions of many-body theories
Source: Nat Commun. 2017 Apr 5;8:14926. doi: 10.1038/ncomms14926 (PMC5382319; doi:10.1038/ncomms14926)
Supplement: Supplementary Information — Supplementary Figures, Supplementary Notes and Supplementary References [file ncomms14926-s1.pdf]

Optimal free descriptions of many-body theories

Supplementary Note 1: Convergence of Ising single body levels. Regarding the rate of convergence to the optimal free description with L, we find that the single particle energies $\{\epsilon\}$ converge exponentially fast to their asymptotic values, as shown in Supp. Fig. 1. This is the case even near criticality due to the finite size induced gap. In the thermodynamic limit, however, the entanglement spectrum is gapless at criticality [1]. Hence the convergence to the optimal model is expected to be polynomial in L. We observe that a power law convergence can be well fitted around criticality, with the goodness of the fit decreasing away from it (data not shown).

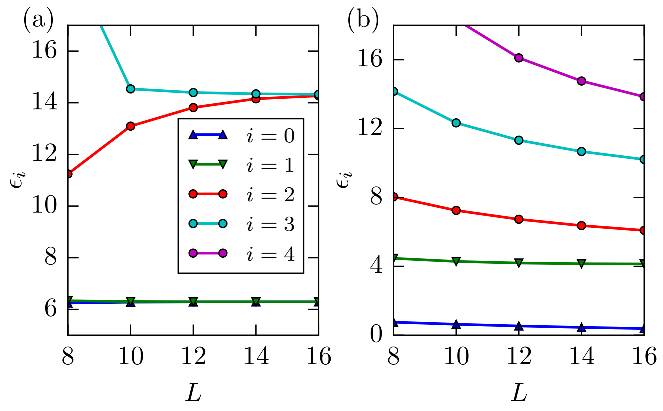

Supplementary Figure 1: **Convergence of the lowest lying single particle entanglement energies.** Graph (a) shows the single-particle entanglement energies ϵ_i of the ferromagnetic model and graph (b) for the antiferromagnetic model as a function of L at the non-critical generic point ($h_z = 0.88$, $h_x = 0.16$). All low lying energies are exponentially converging with L to their asymptotic values.

Supplementary Note 2: Maximum interaction distance among rank-4 entanglement spectra. Consider a general 4-level entanglement spectrum of a normalised ρ which is expressed in terms of the probabilities $\{p\} = \{p_1, p_2, p_3, p_4\}$. The probabilities are ordered to satisfy the constraints

$$p_1 \geq p_2 \geq p_3 \geq p_4, \qquad p_1 + p_2 + p_3 + p_4 = 1. \tag{1}$$

We begin by parametrising the probabilities corresponding to 2-mode (equivalently rank-4) free entanglement spectra by z, a, b with one parameter (we here pick b) fixed by normalisation.

Because the probability spectrum has a definite ordering $z \geq za \geq zb \geq zab$ for any z, a, the cost function can be written in a simple algebraic form,

$$2D(\{z,a\},\{p\}) = |z - p_1| + |za - p_2| + |zb - p_3| + |zab - p_4|\,. \tag{2}$$

The interaction distance is a minimisation of this cost function,

$$D_{\mathcal{F}}(\rho) = \min_{z,a} D(\{z,a\},\{p\}). \tag{3}$$

We can fix an element of the variational class $\mathcal{F}$ by choosing $z = p_1$ and $a = p_2/p_1$ which then forms an upper bounding surface on $D_{\mathcal{F}}(\rho)$ over $\{p\}$,

$$D_{\rm B}(\rho) = D(\{p_1, p_2/p_1\},\{p\}) \geq D_{\mathcal{F}}(\rho). \tag{4}$$

Using the normalisation constraint

$$b = \frac{1}{z(1+a)} - 1 = \frac{1}{p_1 + p_2} - 1, \tag{5}$$

we can simplify the upper bound surface to

$$2D_{\rm B}(\rho) = |zb - p_3| + |zab - p_4| \tag{6}$$

$$= 2\left|\frac{p_1}{p_1+p_2} - p_1 - p_3\right| \tag{7}$$

We are now interested in finding the maximum of $D_{\rm B}(\rho)$ with respect to $\{p\}$ with a view to bounding the maximum of $D_{\mathcal{F}}(p)$. We simplify this problem by instead considering the square $D^2_{\rm B}(\rho)$ which is easier to manipulate. The square is a monotone increasing function, therefore we can equivalently maximise $D^2_{\rm B}(\rho)$ to find the maximum of $D_{\rm B}(\rho)$.

To solve this constrained maximisation, we take derivatives first with respect to p_3,

$$\left(\frac{\partial D^2_{\rm B}}{\partial p_3}\right)_{p_1,p_2} = -2\left(\frac{p_1}{p_1+p_2} - p_1 - p_3\right). \tag{8}$$

The only extremal point of the unconstrained problem is a minimum, therefore when D_B is maximised p_3 must saturate its constraints. Since the lower bound $p_3 = 0$ is that of a rank-2 spectrum which has $D_{\mathcal{F}} = 0$ the maximum is found for $p_3 = p_2$,

$$\left(\frac{\partial D^2_{\rm B}}{\partial p_2}\right)_{p_2=p_3,\, p_3} = -2\left(1 + \frac{1}{(p_1+p_2)^2}\right) \times \left(\frac{p_1}{p_1+p_2} - p_1 - p_2\right). \tag{9}$$

This derivative vanishes for $p_1/(p_1+p_2) - p_1 - p_2$ for which $D_{\rm B} = 0$ and therefore minimal. Hence to maximise $D_{\rm B}$, p_2 must saturate its constraints. At its lower bound $p_2 = 0$ we find $D_{\rm B}(p) = 0$ which cannot be the maximum, hence the maximum is found for $p_2 = p_1$,

$$\left(\frac{\partial D_{\mathrm{B}}^2}{\partial p_1}\right)_{p_1=p_2=p_3} = -4\left(\frac{1}{2} - 2p_1\right)^2. \qquad (10)$$

Because once again the only stationary point is a minimum, the constraints on p_1 must be saturated. The lower bound on p_1 is $1/4$ and the upper bound is $1/3$. Because $p_1 = 1/4$ gives $D_{\mathcal{F}}(p) = 0$, the maximum is found for $p_1 = p_2 = p_3 = 1/3$ and by normalisation $p_4 = 0$.

Substituting these values for $\{p\}$ in Supp. Eq. (4) we have $D_{\mathrm{B}}(1/3, 1/3, 1/3, 0) = 1/6$. Then by direct analytic calculation of $D_{\mathcal{F}}(\{1/3, 1/3, 1/3, 0\})$ one can show that this upper bound is attained. The conclusion is that amongst rank-4 probability spectra $\{1/3, 1/3, 1/3, 0\}$ is the unique maximum of $D_{\mathcal{F}}$ achieving $D_{\mathcal{F}} = 1/6$.

Supplementary References

[1] Calabrese, P. & Lefevre, A. Entanglement spectrum in one-dimensional systems. *Phys. Rev. A* **78**, 032329 (2008).
